# A microfluidic dosimetry cell to irradiate solutions with poorly penetrating radiations: a step towards online dosimetry for synchrotron beamlines

**DOI:** 10.1107/S1600577521002691

**Published:** 2021-04-19

**Authors:** Lucie Huart, Christophe Nicolas, Marie-Anne Hervé du Penhoat, Jean-Michel Guigner, Charlie Gosse, Jérôme Palaudoux, Stephane Lefrançois, Pascal Mercere, Paulo Dasilva, Jean-Philippe Renault, Corinne Chevallard

**Affiliations:** a Université Paris-Saclay, CEA, CNRS, NIMBE, 91191 Gif-sur-Yvette, France; b IMPMC, Sorbonne Université, UMR CNRS 7590, MNHN, 75005 Paris, France; c Synchrotron SOLEIL, 91 192 Saint Aubin, France; d Institut de Biologie de l’Ecole Normale Supérieure, ENS, CNRS, INSERM, PSL Research University, 75005 Paris, France; e LCPMR-UMR7614, Sorbonne Université, 75005 Paris, France

**Keywords:** water radiolysis, dosimetry, soft X-rays, microfluidics

## Abstract

Synchrotron radiation can induce sample damage, whether intended or not. In the case of sensitive samples, such as biological ones, modifications can be significant. To understand and predict the effects due to exposure, it is necessary to know the ionizing radiation dose deposited in the sample. In the case of aqueous samples, deleterious effects are mostly induced by the production of reactive oxygen species via water radiolysis. These species are therefore good indicators of the dose. Here the application of a microfluidic cell specifically optimized for low penetrating soft X-ray radiation is reported. Sodium benzoate was used as a fluorescent dosimeter thanks to its specific detection of hydroxyl radicals, a radiolytic product of water. Measurements at 1.28 keV led to the determination of a hydroxyl production yield, *G*(HO^**.**^), of 0.025 ± 0.004 µmol J^−1^. This result is in agreement with the literature and confirms the high linear energy transfer behavior of soft X-rays. An analysis of the important parameters of the microfluidic dosimetry cell, as well as their influences over dosimetry, is also reported.

## Abstract 2

The application of a microfluidic cell, specifically optimized for low penetrating soft X-ray radiation, as a dosimetry cell is reported. An analysis of the important parameters of the microfluidic cell, as well as their influences over dosimetry, is also reported. Measurements at 1.28 keV led to the determination of a hydroxyl production yield, *G*(HO^**.**^), of 0.025 ± 0.004 µmol J^−1^.

## 1. Introduction   

Damage to samples during exposure to radiation remains a critical issue in many areas of synchrotron research. With the advent of fourth-generation synchrotrons providing a higher brightness (Winick, 1997 ▸; Garman & Weik, 2017 ▸; Henderson, 1995 ▸; Glaeser, 1971 ▸; Teng & Moffat, 2000 ▸), new strategies are needed to avoid such damage becoming disruptive to the measurements quality. These strategies rely on the observation that the damaging effects are not directly linked to the amount of radiation passing through the material at a given point, but more to the energy absorbed by the material (Berger, 1961 ▸). Accordingly, quantities such as the beam intensity or the sample exposure time are not sufficient to fully infer the extent of the damage, but rather appear as useful parameters, among others, for its determination. As a matter of fact, controlling the sample damage implies mastering four essential parameters: (i) the spatial and (ii) spectral distribution of the synchrotron beam on the sample, (iii) the photon flux, and (iv) the delivered dose. In the context of synchrotron radiation, the first three parameters are generally well characterized. On the contrary, the latter parameter remains more difficult to access as it represents the amount of energy absorbed per unit mass of irradiated material. Its value, denoted *D*, is generally given in Gray (1 Gy = 1 J kg^−1^) and depends on the physical characteristics of both the beam and the irradiated sample.

Calorimetry was the primary standard method used to perform dosimetry, *i.e.* to quantify the dose delivered to matter by ionizing particles (Callendar, 1910 ▸). The concept is simple since the measured rise in temperature, following the energy-to-heat conversion, is the most direct consequence of energy absorption by the material. However, in aqueous solution, a part of the absorbed photon energy will actually initiate water radiolysis reactions. Therefore, the measured temperature rise can be greater or smaller than the value of a complete energy conversion, depending on the exo- or endothermic nature of these reactions (Ross & Klassen, 1996 ▸). New developments, based on graphite calorimeters, are currently in progress, but calorimetry remains a cumbersome method for performing dosimetry in liquids (Kim *et al.*, 2017 ▸). As a consequence, to date, chemical dosimetry is the most commonly used technique in radiation research. It can be based on any substrate, called a dosimeter, that undergoes a quantifiable chemical change under irradiation. In order to facilitate measurement, the response of this substrate should be proportional to the dose deposited over a range as wide as possible (dose to be measured can vary from the centigray to the megagray range) and be temperature-independent (Spinks & Woods, 1976 ▸). Other criteria could be considered, such as stability, ease of use or preparation, and stability of response to small changes in the chemical environment. In practice, no dosimeter meets all these criteria, but some come close (Table 1 ▸).

The wide variety of chemical dosimeters reflects the will to select the dosimeter allowing to achieve the highest measurement accuracy for given irradiation conditions. The dosimeter must actually be chosen not only on the basis of its similarity to the sample being studied but also taking into account the characteristics of the beam. The dose deposited in the sample upon exposure to a synchrotron beam is particularly challenging to characterize with a single dosimeter, given the large range of energy (from EUV to hard X-ray) and high flux associated with these micrometre-sized beams. The development of a general method therefore requires setting up a dosimetry system whose sensitivity range can be adjusted. Water-equivalent plastic scintillation detectors are generally used for hard X-ray beam dosimetry (over 100 keV) but require further optimization in the soft X-ray region owing to the dense production of secondary electrons, which interfere with the luminescence signal (Beddar *et al.*, 1992 ▸; Archer *et al.*, 2018 ▸; Ejima *et al.*, 2020 ▸).

**Figure 1:**
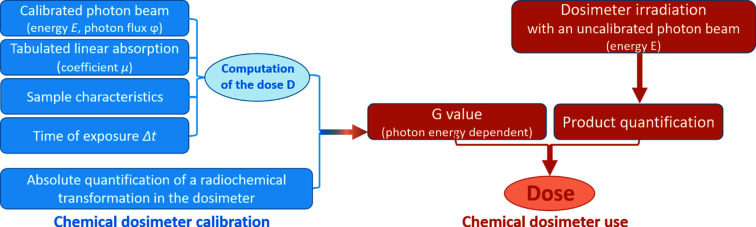
Block diagram showing the main steps of liquid chemical dosimetry. The first step (blue) aims to characterize the dosimeter behavior, *i.e.* to determine the production yield (*G*) of the radio-induced dosimeter’s modification upon exposure to the beam. It therefore requires a good knowledge of both the beam and the sample. The second step (orange) consists of using the calibrated dosimeter to determine the dose from the quantification of the dosimeter’s change in response to the irradiation.

The principle of dosimetry using liquid chemical dosimeters is summarized in Fig. 1 ▸. The first step aims to characterize the dosimeter behavior, *i.e.* to determine the *G*(*X*) values, that correspond to the amount of radiochemical product *X* formed per unit of energy deposited in the solution. This *G*-value, which is photon-energy dependent, can be expressed in molecules per 100 eV energy absorbed, or in mole per Joule,
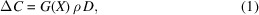
where Δ*C* is the concentration of the radiochemically produced species, directly related to the measured signal, and ρ is the volumetric mass density of the dosimeter solution. A calibrated beam of photons is used so as to finely control the dose delivered to the solution. The accurate quantification of the dosimeter signal as a function of the dose delivered will then allow the calibration of the dosimeter. The second step corresponds to the determination of the dose delivered to the solution upon irradiation with an uncalibrated photon beam. The dose will be determined by measuring the signal of the dosimeter upon exposure. The value of the dose can be determined according to the previously determined *G* value without the need for information on the beam. A comparison with other dosimetry methods will provide a validation of the results.

Among those presented in Table 1 ▸, the best known aqueous dosimeter is the Fricke dosimeter (Fricke & Hart, 1935 ▸), based on the oxidation of ferrous sulfate ions. Its *G*-value (Fig. 1 ▸), corresponding here to *G*(Fe^3+^), was experimentally determined for various radiation exposures (Lazo *et al.*, 1954 ▸; Watanabe *et al.*, 1995 ▸; Hoshi *et al.*, 1992 ▸; LaVerne & Schuler, 1987 ▸; Klassen *et al.*, 1999 ▸). However, similar results in the soft X-ray region (<2 keV) remain poorly reported to date (Vyšín *et al.*, 2020 ▸). Indeed, due to the high absorption coefficient of liquid water in this region, low-energy photons penetrate only a few tens of micrometres. This leads both to a low average dose rate (dose per unit of time) and to a heterogeneous dose deposition on macroscopic samples. The dose determination thus appears very challenging for samples of millimetre thickness or more.

We recently demonstrated the use of the benzoate system in liquid samples upon soft X-ray exposure (Huart *et al.*, 2020 ▸). This dosimeter has been shown to allow a very sensitive detection of the hydroxyl radicals produced by water radiolysis under these specific conditions. Hydroxylation of benzoate ions leads to the formation of fluorescent species, namely hydroxy-benzoate ions, that can be easily detected even at low dose (Armstrong *et al.*, 1960 ▸; Musat *et al.*, 2010 ▸).

Our first measurements were performed using a 1 cm-thick static cell. To overcome the inhomogeneous dose deposition in this cell, it is replaced by a microfluidic cell in the reported study. This device provides a reduced and well defined sample environment leading to a more homogeneous irradiation of the solution. Additionally, microfluidics offers many advantages as it allows modulating the dose by simply varying the flow rate, and enables the sequential injection of samples and the control of dissolved gasses. We report here its successful implementation in the most demanding case of low-penetrating radiation (1.28 keV soft X-rays). The results presented suggest the great versatility and potential adaptability of the device to a large variety of radiations.

## 2. Materials and methods   

### 2.1. Chemical dosimeter   

Chemicals of sodium benzoate (NaBz) and sodium 2-hydroxy-benzoate (Na2HB) were purchased from Sigma Aldrich and used as received. Dosimeter solutions of concentrations in the 0.01 to 1 *M* range were prepared by dissolving the NaBz salt (purity ≥ 99.6%) in water. Solutions were freshly prepared using MilliQ water (of resistivity 18.2 Ω.cm and with less than 10 p.p.b. of carbon organic content) resulting in a measured pH of 8.1. Non-irradiated NaBz solutions most often present a residual fluorescence emission due to partial hydroxylation with time (see Fig. S1 of the supporting information). Therefore, the benzoate solution was chosen with regard to its low fluorescence emission, in order to minimize the fluorescence background.

Under irradiation, aromatic hydroxylation occurs pre­dominantly at the ortho-position (Armstrong *et al.*, 1960 ▸). Moreover, 2-hydroxy-benzoate (2HB) shows a high fluorescence under these conditions of pH (8.1–8.8) whereas 4-hydroxy-benzoate fluoresces at lower wavelengths and 3-hydroxy-benzoate is only fluorescent when doubly deproton­ated, at basic pH (Musat *et al.*, 2010 ▸; Armstrong *et al.*, 1960 ▸). Known amounts of Na2HB (purity ≥ 99.9%) were added to NaBz solutions to calibrate the dosimeter. The concentration of Na2HB ranged from 10^−7^ to 10^−4^ *M*. Emission spectra were recorded in a quartz suprasil cell (Hellma Analytics^®^, Art. 101-057-40) using a FluoroMax-4 spectrometer from Horiba^®^ (Kisshoin, Japan) with λ_excitation_ = 300 nm and λ_emission_ = 420 nm (see Fig. S1). Calibration curves for the emitted fluorescent signal (*F*) can be fitted by using a linear regression, 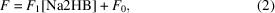
where *F*_1_ is the slope and *F*_0_ the residual fluorescence. *F*_0_ may vary due to aging of the solution and should be measured regularly in order to best characterize the evolution of the irradiated sample. An inner-filter effect, *i.e.* a partial absorption of both the excitation and emission signals by the solution, affects the 2HB fluorescence signal for the 1 *M* NaBz solution, which leads to a slower, but still linear, increase of the fluorescence signal with the Na2HB concentration (see Fig. S1).

### 2.2. Microfluidic dosimetry cell   

**Figure 2:**
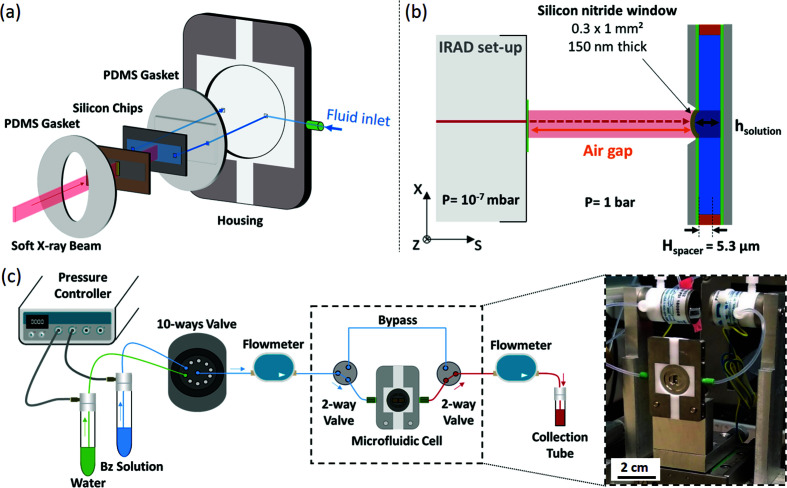
Experimental setup used for soft X-ray beam irradiation. (*a*) Exploded view of the microfluidic cell. (*b*) Not-to-scale cross-section diagram of the irradiation setup (*s* represents the synchrotron axis). (*c*) Diagram of the fluid actuation system and photograph of the microfluidic cell mounted on the Metrology beamline at SOLEIL.

The dosimetry cell, containing the liquid to be irradiated, was constructed by adapting the design of a microfluidic cell originally developed for soft X-ray spectro-microscopy in liquids (Gosse *et al.*, 2020 ▸). It consists of two silicon chips (Silson, Southam, UK) assembled in a home-made fluidic housing that allows the liquid to flow between them (see Fig. 2 ▸). The original microfluidic cell developed by Gosse and collaborators has only been modified with respect to the chip characteristics (membrane size, spacer material and thickness – see below), while all other elements (chip housing, gaskets, fluid control system and accessories) have remained essentially unchanged. All materials used have been chosen to ensure total chemical inertness of the microfluidic system. Both silicon chips are 200 µm thick and 3 mm × 6 mm in size. The Si chip facing the X-ray beam supports at its center a low stress (≤250 MPa) silicon nitride (Si_3_N_4_) membrane, which is 150 nm thick and 0.3 mm × 1 mm large. This membrane is highly transparent to soft X-ray, with transmission values between 91.2 and 96.4% over the energy range from 1.0 to 1.4 keV (Henke *et al.*, 1993 ▸). In addition, this chip carries a rectangular hollow spacer, made of SU-8 photoresist, which has a thickness of *H*_spacer_ = 5.30 ± 0.05 µm. This spacer delimits the volume of liquid enclosed between the chips during the experiment. The latter is 1.2 mm wide, 5 mm long and 5.30 µm thick and therefore is equal to 32 nL in the absence of membrane deformation. The other silicon chip has two side holes (0.1 mm × 0.1 mm), which allow the fluid to flow through the microchannel of the cell. The sandwich of silicon chips is surrounded by two home-made polydimethylsiloxane (PDMS) gaskets (see Fig. 2 ▸) that ensure the complete sealing of the microfluidic cell up to 2 bar. The entire system is inserted in a polytetrafluoroethylene (PTFE)/metal housing (Gavard, Arrou, France) fully described in our previous paper (Gosse *et al.*, 2020 ▸). It carries polyetheretherketone (PEEK) fluid fittings (IDEX Health Science, Oak Harbor, USA) for fluid injection. Fluorinated ethylene-propylene (FEP) and PEEK tubings with an inside diameter of 0.01 inch are used to connect the microfluidic cell to the fluid injection system.

The fluidic actuation is based on a pressure regulator (MFCS-EZ, Fluigent, Le Kremlin-Bicêtre, France), supplied with compressed air, which allows to precisely adjust the gas pressure above the solution to be injected. This results in an actuating pressure difference, Δ*P*, between the inlet and outlet of the fluidic system in the range of 0 to 2 bar. Flowmeters are located downstream and upstream of the dosimetry cell, as presented in Fig. 2 ▸. Two different flowmeter unit (S or M type, Fluigent) were used to allow flow measurements in the range 0.42–80 µL min^−1^ with an accuracy of 5%. The solution to be injected is selected by means of a ten-way bi-directional valve (M-switch, Fluigent), which allows the sequential injection of up to ten different solutions. This is particularly useful for conditioning the microfluidic cell, which requires first injecting ethanol for pre-wetting the system, then water, and finally the solution to be irradiated. Two bi-directional two-way valves (2-switch, Fluigent) allow the fluid to bypass the cell and therefore to renew most of the solution in the fluidic system in a timely manner. The hydrodynamic resistance of the cell, about 4 × 10^14^ Pa s m^−3^, is indeed one order of magnitude greater than the one of the bypass capillary (3 × 10^13^ Pa s m^−3^). As a result, the flow rates achievable in the bypass path are approximately one order of magnitude higher than those achievable in the cell path. The selection of the cell path, in a second step, completes the fluid renewal in the microfluidic system. The flow rate in the microchannel of the cell is typically 13 µL min^−1^ when injecting pure water at Δ*P* = 1000 mbar.

### 2.3. Synchrotron-based soft X-ray irradiations   

#### 2.3.1. Characterization of the soft X-ray beam   

The beam extraction setup (IRAD) (Hervé du Penhoat *et al.*, 1999 ▸, 2010 ▸), initially developed for the LURE synchrotron, was adapted to the soft X-ray branch of the Metrology beamline at Synchrotron SOLEIL (Saint-Aubin, France) in order to allow irradiation in air (Huart *et al.*, 2020 ▸). Briefly, the photon beam is extracted from the vacuum environment of the beamline thanks to a differential pumping system, terminated by a square 1 mm × 1 mm large and 150 nm-thick silicon nitride window (FASTEC, Northampton, UK). The energy of the beam used for the irradiation experiments was 1.28 keV corresponding to a photon flux of φ = 8.61 × 10^9^ photons s^−1^ at the entrance of the microfluidic cell Si_3_N_4_ membrane. A 0.4 µm-thick boron filter was used to ensure that the contribution of higher harmonics to the measured dose was less than 1% (Huart *et al.*, 2020 ▸).

**Figure 3:**
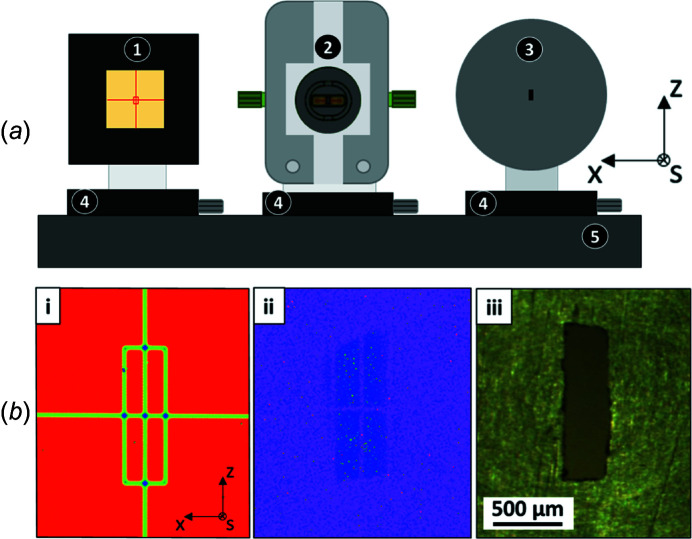
(*a*) Scheme of the devices mounted in front of the beam: 1 – camera with engraved YAG:Ce scintillator; 2 – microfluidic dosimetry cell; 3 – masked photodiode; 4 – manual micro-translation table (*s*-axis); 5 – AXMO translation table (*x*, *z*-axis). (*b*-i) Photograph of the scintillator with engraved axes and rectangle pattern to simulate the microfluidic window (see text for more details). (*b*-ii) Visualization of the synchrotron beam projected on the YAG target (in blue). The width of the monochromator exit slits was adjusted to 500 µm (*z*-axis), resulting in a beam almost similar to the vertical dimension of the cell window. (*b*-iii) Photograph of the brass mask applied on the photodiode: a rectangle 0.29 mm × 1.07 mm was drilled in the center with a precision of 20 µm.

Conducting irradiation experiments requires precise positioning of the sample in front of the X-ray beam and therefore a fine characterization of the beam spatial extension. It is all the more crucial when the beam is of microscopic size and when the sample to be aligned is contained in a microfluidic channel. Two detecting devices, namely a YAG:Ce scintillator crystal and a photodiode, were used to determine the beam geometry, as well as the incoming photon flux. The two detectors were placed on either side of the dosimetry cell, on a common *x–z* translation table (AXMO, MNT9 model, 0.2 µm precision, Brétigny/Orge, France) (see Fig. 3 ▸). Prior to mounting the entire setup on the beamline, relative positions of the two detectors, with respect to the center of the microfluidic dosimetry cell, were measured using an optical level (Wild Heerbrugg, Heerbrugg, Switzerland). Each device was additionally mounted on a one-direction manual translation stage (*s*-axis, see Fig. 3 ▸). Once the entire setup installed on the beamline, these stages allow one to independently adjust the air gaps, *i.e.* the distance between the detecting devices and the IRAD exit window [Fig. 2 ▸(*b*)]. Due to the specific housing associated with each device, the exact air gaps were 2.2 mm for the microfluidic dosimetry cell, 3.06 mm for the photodiode and 1 mm for the YAG:Ce.

The first of the two detectors consists of a 50 µm-thick scintillator crystal (YAG:Ce from Crytur, Turnov, Czech Republic) installed in front of a CCD camera (acA2500-20gm, Basler, Ahrensburg, Germany) in order to visualize the shape of the extracted beam by converting X-ray photons to visible light. The horizontal (*x*) and vertical (*z*) axes, as well as a rectangle with the dimension of the Si_3_N_4_ window (0.3 mm × 1 mm), were etched on the scintillator crystal surface to provide a spatial reference [Fig. 3 ▸(*b*-i)]. The exit slits of the beamline monochromator were adjusted to 500 µm in order to increase the beam width along the *z*-axis. Due to the magnification factor of the beamline, the resulting beam had a vertical size close to the cell window height (1 mm). The use of the YAG crystal showed that increasing even more the slit opening had no effect on the vertical beam dimension, since the beam was then intercepted by the diaphragms of the differential pumping stage [see the rounded shape of the upper side of the beam in Fig. 3 ▸(*b*-ii)]. Horizontal broadening is determined by the optical characteristics of the beamline.

On the other side, a photodiode (AXUV-100G Ti/C from International Radiation Detector, Torrance, California, USA) was used to characterize the beam intensity profile. This photodiode was calibrated with respect to an ionization chamber (Boissière, 2004 ▸). As the photodiode delivers intensity values integrated over the whole photosensitive area, it was equipped with a brass mask featuring a central rectangular hole with dimensions almost similar to those of the dosimetry cell window. This system thus makes it possible to quantify the intensity actually received by the sample [Fig. 3 ▸(*b*-iii)]. A scan of the photodiode along the vertical *z*-axis allowed us to estimate the vertical beam width to be approximately 670 µm (Fig. S4). This result is consistent with the observations performed using the YAG crystal, which showed that the beam was smaller than 1 mm (Fig. 3 ▸). Results along the horizontal *x*-axis were used to retrieve the characteristics (shape, intensity distribution) of the beam, and are presented in Section 3.1. The evolution of the current delivered by the photodiode as a function of the opening of the monochromator slits was also recorded (Fig. S5). The current increases linearly with the slits opening and reaches a plateau around 500 µm. Again, this result is consistent with the observations using the YAG crystal. It testifies to the good alignment of the brass mask and therefore of the microfluidic cell. The beam position remains in principle stable for a selected photon energy, while varying the energy could imply modifying some optical elements of the beamline (gratings or optical filters). Consequently, the characterization of the soft X-ray beam was usually done at the beginning and at the end of the experiment only, so as to check the actual stability of the beam. However, this procedure could be easily repeated in case there was any doubt on the stability of the beam position. In the reported experiments, a fluctuation of the beam intensity was observed (between 0 and 30% over one week of measurement), which was attributed to mechanical relaxation of the setup.

#### 2.3.2. Protocol of irradiation in the microfluidic dosimetry cell   

Prior to irradiation, the entire microfluidic system was washed with 300 µL of fresh solution, the beam shutter being closed. Since this volume corresponds to three times that contained in the system (dosimetry cell, valves and tubing), it ensures that the previous content has been fully rinsed away. The flow rate was next precisely adjusted to provide known, reproducible conditions and the beam shutter was opened. A volume of fluid corresponding to three times the volume of the downstream capillaries was first discarded to make sure that fluorescence measurements were performed on irradiated samples that were free from any dilution by non-irradiated liquid. Finally, approximately 150 µL of irradiated sample were collected in Eppendorf^®^ tubes and stored in the dark at 4°C prior to *ex situ* fluorimetric analysis (see Section 2.1).

### 2.4. Dosimetry based on photodiode measurements and optical profilometry   

The absorbed dose, *D*, is defined as the energy deposited in the system per unit of mass. When irradiating a solution in the microfluidic dosimetry cell, the dose is inhomogeneously deposited in the micrometre-thick film of solution, owing to the exponential decrease of the beam intensity through the film. One can then consider an average dose, which corresponds to the overall dose deposited in the film normalized by the mass of the irradiated solution. This average dose can be calculated using the following equation,
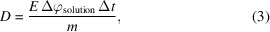
where *E* is the photon energy of the beamline, Δφ_solution_ is the number of photons absorbed per unit of time in the solution, Δ*t* is the exposure time and *m* the mass of sample irradiated. In the microfluidic channel, the flow is a pressure-driven Poiseuille flow with a parabolic profile in both the *s* and *z* directions (Bruus, 2008 ▸). The mean exposure time, Δ*t*, can be expressed in terms of the mean velocity of the fluid passing in front of the beam, which in turn is related to the volumetric flow rate, *Q*. The previous equation becomes 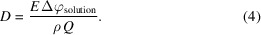
Among the four parameters presented, Δφ_solution_ is the only one which cannot be straightforwardly measured. However it can be deduced from the flux measured at the photodiode surface (φ_photodiode_) taking into account the absorption properties of the different materials the beam goes through (Owen *et al.*, 2009 ▸),
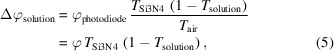
where *T*_Si3N4_ is the transmission factor through the Si_3_N_4_ cell window, *T*_solution_ the one of the solution in the microfluidic channel and φ is the photon flux at the entrance of the microfluidic cell (see Section 2.3.1). *T*_air_ is the transmission factor resulting from the 0.86 mm difference of air gap between the photodiode and the dosimetry cell (see Section 2.3.1). The transmission coefficient of a pure material of thickness *h* is given by *T* = exp[−μ(*E*)*h*], where μ is the linear attenuation coefficient of the material, which can be calculated from the elemental absorption coefficients at a given photon energy (Henke *et al.*, 1993 ▸). At 1.28 keV, *T*_Si3N4_ = 0.954 for a membrane with 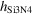 = 150 nm [density of 2.68 ± 0.16 g cm^−3^ (Huszank *et al.*, 2016 ▸)]. The transmission factor for an air gap difference of 0.86 ± 0.16 mm is *T*_air_ = 0.825. Finally *T*_water_ = 0.324 for a water film of thickness *h*_solution_ = *H*_spacer_ = 5.3 µm. *T*_solution_ values were in fact calculated taking into account the exact nature of the solution circulating in the cell, as well as the actual thickness of the microfluidic channel. A systematic analysis of the uncertainties associated with the determination of these transmission values is presented in Section 3.3.

**Figure 4:**
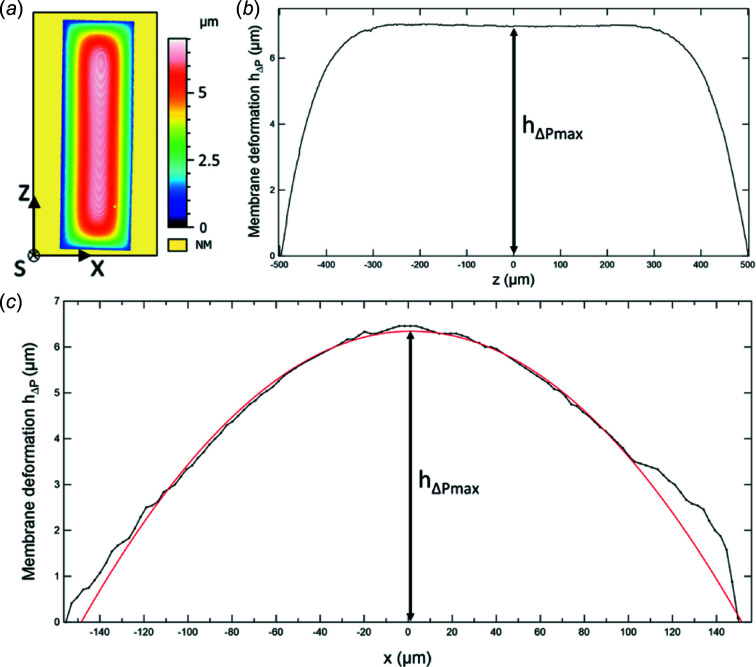
Characterization of the Si_3_N_4_ membrane deformation during the injection of a 0.01 *M* NaBz solution under an actuation pressure difference Δ*P* = 800 mbar. (*a*) Profilometry scan providing a 2D map of the bulging. (*b*) Deformation profile along the *z*-axis. (*c*) Deformation profile along the *x*-axis: raw data (black symbols) and parabolic fit (red line). Maximum deformation is defined as the top value of the parabola (black arrow).

Under the hydrodynamic pressure induced by fluid flow, the Si_3_N_4_ window experiences some bulging, so that *h*_solution_ becomes greater than *H*_spacer_ almost everywhere (see Fig. 2 ▸). This bulging increases the number of photons absorbed by the irradiated sample and therefore the average dose deposited in the sample. The exact determination of *T*_solution_ requires the experimental measurement of the dosimetry cell deformation in flow conditions (*Q* > 0, see Section 3.2). The deformation of the membrane was thus carefully characterized, before and after the irradiation experiments, using an optical interferometer (smartWLI Prime, Schaefer Technologie GmbH, Langen, Germany) equipped with a 20× Mirau objective. Typical profilometry results are presented in Fig. 4 ▸. By scanning the Si_3_N_4_ window at a given flow rate *Q*, *i.e.* a given actuation pressure difference Δ*P*, the profilometer provides a 2D map of the bulging [Fig. 4 ▸(*a*)]. The transverse deformation profile at the center of the membrane (*h*_solution_ = *H*_spacer_ + *h*_Δ*P*_) can in particular be extracted from this map. The profile along the *z*-axis testifies to the homogeneous deformation of the membrane. Along the *x*-axis [Fig. 4 ▸(*c*)], the profile can be fitted with a parabolic function in first approximation (Holtz *et al.*, 2013 ▸; Small & Nix, 1992 ▸). It allows determining the maximum deformation value *h*_Δ*P*_ _max_ = *h*_Δ*P*__(*x*=0,*z*=0)_. Δφ_solution_ was computed using the two extreme values for *h*_solution_ (*i.e.* *H*_spacer_ and *H*_spacer_ + *h*_Δ*P*_ _max_), and the average value was used in dose calculations. The errors induced by this approximation were cautiously considered during the analysis of the results (see Section 4).

In addition, equation (5) assumes that the photon beam passing through the window of the dosimetry cell is identical to the one going through the hole of the photodiode mask, once its intensity has been corrected to take into account the air gap difference and the size of the mask opening. It is therefore essential to center precisely both devices with respect to the photon beam, not only to maximize the dose deposition in the solution but also to enable an accurate calculation of the dose based on the photodiode measurements. As discussed in Section 2.3, the vertical beam dimension was smaller than the length of both the hole in the brass mask and the microfluidic Si_3_N_4_ window. The *z*-axis alignment was therefore straightforward. On the contrary, the alignment along the *x*-axis was particularly challenging owing to the fact that the horizontal extension of the beam is larger than the cell window, as illustrated in Fig. 3 ▸(*b*-ii). Centering was first achieved by using the known relative positions of the photodiode and YAG crystal with respect to the dosimetry cell, and was next optimized using fluorescence measurements (see Section 3.1).

## 3. Results   

### 3.1. Response of the flowing dosimeter to soft X-ray exposure   

We conducted series of irradiation at a fixed photon energy *E* (1.28 keV) while varying the position of the microfluidic dosimetry cell in front of the beam. Using equations (1), (2) and (4), one can show that the fluorescence signal (*F*) of the irradiated NaBz solution is directly proportional to Δφ_solution_. It obeys equation (6),
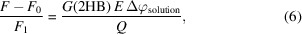
where *G*(2HB) is the production yield of 2HB species formed during irradiation and parameters *F*_0_ and *F*_1_ refer to the calibration curves (see Fig. S1). *F*_0_ was preferentially extracted from dose-fluorescence response curves (extrapolated value at zero dose). At a fixed flow rate, the fluorescence signal only varies according to the absorbed photon intensity Δφ_solution_ that is directly proportional to the incident beam photon flux φ. Two different batches of 0.01 *M* NaBz solutions were successively considered to detect potential reproducibility issues.

The curves representing the normalized fluorescence intensity for the two different flow rates fully overlap and can be well fitted by Gaussian functions with full width at half-maximum (FWHM) equal to 342 ± 17 µm for *Q* = 8 µL min^−1^ and 336 ± 15 µm for *Q* = 5 µL min^−1^. The vertical error bars were calculated considering the dispersion of the fluorescence calibration curves (Fig. S1). In the fitting procedure, these errors were taken into account using an orthogonal distance regression fitting method.

**Figure 5:**
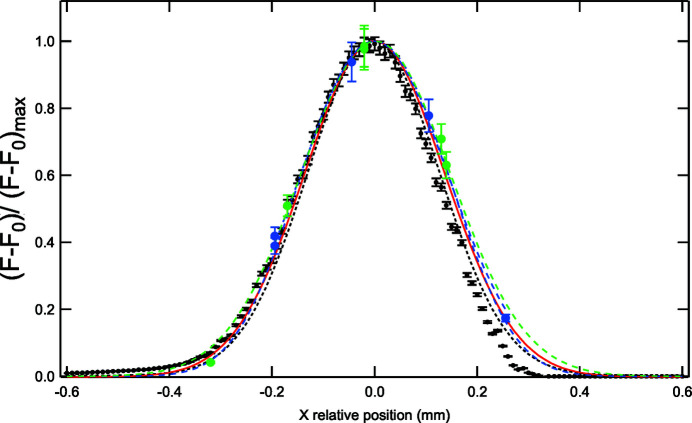
Normalized fluorescence signal resulting from the irradiation of two batches of 0.01 *M* benzoate solution, in the microfluidic dosimetry cell, by a soft X-ray beam at 1.28 keV (green symbols: 8 µL min^−1^; blue symbols: 5 µL min^−1^). The photon flux measured with the masked photodiode for various *x*-positions of the photodiode is displayed for comparison (black symbols). Gaussian fits of the experimental data are also represented (dotted lines). The red plot corresponds to the convolution of a rectangle function, with a width of 0.3 mm, by a Gaussian function with a FWHM of 230 µm.

In parallel, the electrical current at the masked photodiode was recorded for various positions of the photodiode along the *x*-axis, in order to reconstruct the beam intensity profile in this direction (Fig. 5 ▸, black symbols). Again, the obtained curve can be fitted by a Gaussian and its FWHM was found to be 303 µm. Deconvoluting this Gaussian function by a 270 µm-wide rectangle function (corresponding to the width of the hole in the photodiode mask) allowed the determination of the actual beam profile. This results in a Gaussian profile of 230 µm FWHM. It was then possible to model the intensity of the beam passing through the entrance Si_3_N_4_ window of the dosimetry cell by convoluting the beam profile with a 300 µm slit. The resulting curve exhibits a FWHM of 321 µm (Fig. 5 ▸, red curve). This result is in full agreement with the Gaussian function fitted to the normalized fluorescence signal, with a difference between the FWHMs of less than 10%. These measurements demonstrate that the fluorescence response of the irradiated dosimeter is fully proportional to the received beam intensity. Since the signal is directly related to dose-dependent water radiolysis, this confirms that over-irradiation, which would translate into nonlinearities, does not affect the dosimetry measurements [equation (6)]. Moreover, these fluorescence measurements can also be used to check the correct positioning of the dosimetry cell in front of the beam. However, at 1.28 keV and a flow rate of 8 µL min^−1^, it takes at least 45 min to collect the 150 µL of irradiated sample necessary for each data point. Therefore, in future experiments this strategy should only be used to validate the cell position, once it has been aligned using the masked photodiode and the scintillator.

### 3.2. Si_3_N_4_ membrane deformation under flow   

Our study focuses on soft X-ray dosimetry, which is particularly challenging owing to the low penetration (only a few micrometres) of this type of radiation in water. In order to maximize the amount of photons that enter the solution, a 150 nm-thick Si_3_N_4_ membrane has been selected as the entrance window of the dosimetry cell. However, such a thin membrane can undergo strong elastic deformations under the hydrodynamic pressure associated with liquid flow (Qiao *et al.*, 2018 ▸; Keskin *et al.*, 2019 ▸; Gosse *et al.*, 2020 ▸). The flow-induced bulging can lead to a substantial change in the received dose. Indeed, the absorption of the solution is much less influenced by the concentration of the benzoate solution than by the sample thickness at 1.28 keV (see Fig. S8). Absorption at this photon energy actually varies from 0.65 to 0.93, that is by 43%, as the sample thickness increases from 5.3 to 12.5 µm, whereas it only varies by less than 1% at 1.28 keV when increasing the benzoate concentration from 0.01 *M* to 1 *M*. Consequently, to perform accurate dosimetry measurements, membrane bulging must be finely characterized.

**Figure 6:**
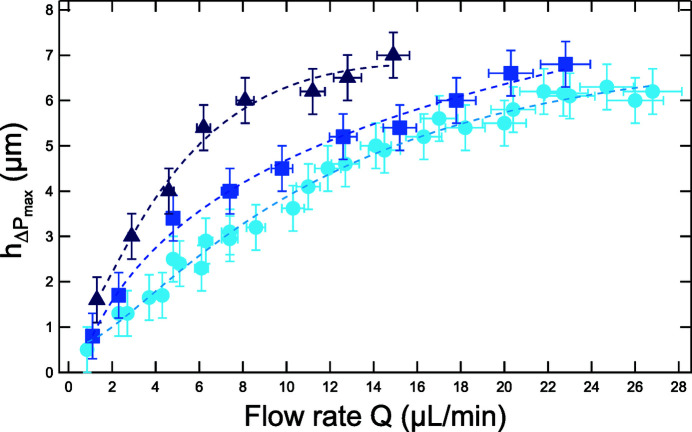
Maximum deformation, as a function of the flow rate, for three different concentrations of the flowing benzoate solution: light to dark blue correspond to 0.01 *M*, 0.1 *M* and 1 *M*. Maximum deformation values are measured with an uncertainty of 0.5 µm. The dotted lines are only a guide to the eye.

The maximum deformation, achieved at the center of the Si_3_N_4_ membrane, was measured using optical interferometry (see Section 2.4). Results are plotted in Fig. 6 ▸ as a function of the flow rate, *Q*, and for three different NaBz concentrations. For the 1 *M* solution, the deformation of the Si_3_N_4_ membrane strongly increases for flow rates up to 8 µL min^−1^, and then reaches a plateau at about 7 µm. A similar behavior, although less marked, is observed for the two other concentrations. It should be noted that irradiation experiments were mainly performed at flow rates lower than 10 µL min^−1^, since at higher flow rates the amount of radiolytic products was too low to be quantified by fluorimetry.

These measurements highlight that, even if the benzoate concentration does not directly influence the absorption factor in the solution at 1.28 keV, it changes the maximum membrane deformation. This can be explained by considering that the increase in the solution viscosity, for larger NaBz concentrations, generates an increased resistance of the system to fluid flow. Therefore, to obtain a given flow rate, one needs to apply a larger actuation pressure difference Δ*P*, which translates into a larger membrane deformation [see Fig. S6(*a*)]. The increase of the experimental viscosity (η) with the NaBz concentration was confirmed by two independent measurements. Firstly, considering the hydrodynamic resistance of the entire fluidic system (*R*_tot_) (Gosse *et al.*, 2020 ▸) and, secondly, using an Oswald viscosimeter [see Fig. S6(*b*)]. Determined values of η were 0.995, 1.004 and 1.106 ± 0.002 mPa s, respectively, for the three solutions tested (0.01 *M*, 0.1 *M* and 1 *M*).

### 3.3. Hydroxyl radicals production yield determination and related uncertainties   

**Figure 7:**
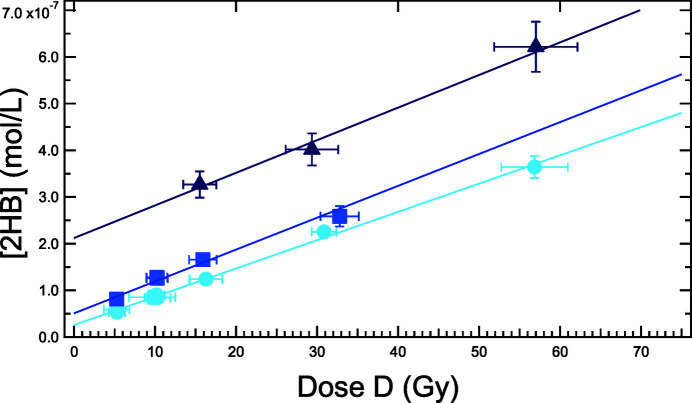
Relationship between the calculated average dose and the 2-hydroxy­benzoate concentration of benzoate solutions, at various concentrations, irradiated by soft X-ray at 1.28 keV: light to dark blue correspond to 0.01 *M*, 0.1 *M* and 1 *M*.

The evolution of the 2HB concentration in irradiated solutions, as a function of the dose value, is presented in Fig. 7 ▸. The hydroxyl radical concentration can be deduced from the efficiency of benzoate hydroxylation, in aerated conditions, reported to be 30% above 0.01 *M* (Loeff & Swallow, 1964 ▸; Armstrong *et al.*, 1960 ▸).

A linear relationship between the absorbed dose and the dosimeter response was shown for all solutions irradiated at 1.28 keV. In each case, the production yield of HO^**.**^ radicals can be deduced from the slope of the linear function that fits the data (Fig. 7 ▸). This slope appears relatively independent of the benzoate concentration and leads to the following *G*(HO^**.**^) values: *G*(HO^**.**^)_0.01*M*_ = 0.024 ± 0.003 µmol J^−1^, *G*(HO^**.**^)_0.1*M*_ = 0.026 ± 0.003 µmol J^−1^ and *G*(HO^**.**^)_1*M*_ = 0.026 ± 0.003 µmol J^−1^. An independent set of measurements, performed a year before on the same beamline on 0.01 *M* NaBz, led to *G*(HO^**.**^)_0.01*M*_ = 0.025 ± 0.004 µmol J^−1^ (see Fig. S9), a very similar value despite the systematic alignment errors and potential aging of benzoate salts. Sources of uncertainties considered for the experimental determination of the production yield are listed in Table 2 ▸. They were estimated at 1.28 keV, but can vary depending on the photon energy.

The uncertainty on the photon intensity (3.3%) was estimated by considering previous measurements performed at the Metrology beamline (Huart *et al.*, 2020 ▸). The one on the air gap transmission (2.2%) was determined from the precision of the device positioning in front of the IRAD setup. The error on relative positioning of the synchrotron beam and sample environment (5.6%) has been assessed by calculating the effect of a 50 µm misalignment. The error on the photon transmission through the Si_3_N_4_ window of the dosimetry cell (1.4%) was estimated according to uncertainties on the stoichiometric composition of the Si_*x*_N_*y*_ material (from Si_3_N_4_ to SiN), its thickness (10%) and on the material density. Reported values for the latter parameter actually vary between 2.68 g cm^−3^ (Huszank *et al.*, 2016 ▸) and 3.1 g cm^−3^ (manufacturer specifications). Still, the largest uncertainty is related to the evaluation of the X-ray transmission coefficient of the solution as this factor is strongly dependent on the hydrodynamic pressure. At high flow rates (≥10 µL min^−1^), this uncertainty can reach 16%, owing to the large deformation of the membrane. Besides, high flow rates will decrease the deposited dose and therefore reduce the accuracy of fluorescence measurements. The benzoate dosimeter (irradiated in the microfluidic dosimetry cell) is therefore more accurate at low flow rates (less than 10 µL min^−1^) where errors due to membrane deformation remain limited. Finally, the error on the fluorescence measurements is deduced from the variations of the dosimeter calibration curves. It is estimated to 6.5% at low benzoate concentration (0.01 *M*) but can reach 8.0% at high concentrations due to fluorescence re-absorption. However, the main factor that impedes the accurate determination of the fluorescence signal is not the systematic error on fluorescence measurements but the residual fluorescence of the non-irradiated solution, which becomes the main source of error of the fluorescence signal when considering concentrated solutions. Since the errors were considered as independent of each other, the total relative error on the hydroxyl production yield was calculated using the quadrature method (Gupta, 2012 ▸). It was estimated to about 15% for a 0.01 *M* benzoate solution circulating at 8 µL min^−1^ in the dosimetry cell and irradiated at 1.28 keV.

The detection of HO^**.**^ radicals is particularly important given their high involvement in radio-biological damage. In this context, several studies have provided results on the radiolytic efficiency of HO^**.**^ in different energy ranges (EUV, soft and hard X-rays, gamma rays) (Table 3 ▸). One can observe that the *G*(HO^**.**^) value highly depends on the linear energy transfer (LET) of the ionizing radiation, which corresponds to the energy loss per unit path length. Particles with high LET induce a very high density of ionizations at the nanometre scale (Fulford *et al.*, 1999 ▸). Highly reactive species like water radicals formed during these dose deposition processes can then react together through intra-track recombination, leading to an apparent production yield that decreases when the LET increases (see Table 3 ▸).

Different X-rays studies, both experimental and theoretical, have shown that a minimum production yield *G*(HO^**.**^) is reached when irradiating at about 1 keV (Chen *et al.*, 2009 ▸; Magee & Chatterjee, 1978 ▸; Fulford *et al.*, 1999 ▸; Yamaguchi, 1989 ▸; Vyšín *et al.*, 2020 ▸; Yamaguchi *et al.*, 2005 ▸). The reported *G*(HO^**.**^) values at 1 keV range from 0.023 µmol J^−1^ to 0.15 µmol J^−1^, probably because of the high ionization density and secondary reactions.

Using the microfluidic dosimetry cell, two sets of measurements conducted to a mean value of *G*(HO^**.**^)_0.01*M*_ = 0.025 ± 0.004 µmol J^−1^. The similarity of these results, recorded one year apart, attests to the robustness of the method. This is particularly promising for dynamic measurement even if these values are in the lower range of the production yields reported in the literature. The system have shown great sensitivity upon soft X-ray beam exposure, even at low deposition dose (≤20 Gy). In addition, the constant value of the production yield over the wide concentration range tested is in full agreement with the high LET behavior of soft X-rays, also observed in static studies (Huart *et al.*, 2020 ▸; Goodhead, 2006 ▸).

## 4. Discussion   

Given its chemical inertness and versatility, the dosimetry environment presented here can easily be adapted to very different photon fluxes by choosing the appropriate chemical dosimeter: benzoate or coumarin in the Gray range, Fricke in the hundreds of Gray range, or cerium in the kGy/MGy range. Sodium benzoate was selected in our study, in preference to the more sensitive coumarin derivatives (Louit *et al.*, 2005 ▸), owing to its low toxicity, and to its chemical stability which allows a long-term storage of the dosimeter (Huart *et al.*, 2020 ▸; Musat *et al.*, 2010 ▸; Armstrong & Grant, 1960 ▸) and an easy use in synchrotron environments.

Besides, the microfluidic environment brings several advantages to the dosimetry process. First, using a 0.3 mm × 1 mm microchannel allows to sample the deposited dose on a submillimetre scale, and therefore to map an irradiation field. Second, the micrometre-sized thickness of the channel provides a high precision on the dose deposition process along the beam axis. Finally, the use of continuous flow conditions for the irradiation prevents an over-irradiation of the dosimeter by continuously renewing the sample under the beam. Over-irradiation, which can affect all chemical dosimeters (Pikaev, 1972 ▸), leads to inconsistent measurements and is therefore a major concern in dosimetry. With synchrotron radiation, owing to the high incident photon flux strong over-irradiation of the dosimeter may occur. Indeed, when the dosimetry cell was used without fluid circulation, irreproducible results were obtained (data not shown). It should be noted that, when using a 1 cm-thick static cell, over-consumption of the dosimeter is prevented thanks to the diffusion of both benzoate and dissolved oxygen along the beam axis (Huart *et al.*, 2020 ▸). Here, the observed linear relationship between the fluorescent signal and the calculated dose shows that inducing a continuous flow of dosimeter solution in an appropriate flow rate range is effective in preventing over-irradiation issues. Furthermore, precisely controlling the flow rate allows selecting the dose range that best matches the optimal sensitivity range of the chemical dosimeter. Finally, the flow rate range achievable with our microfluidic design (1–28 µL min^−1^) is another advantage of this dosimetry setup, as it is largely compatible with several in-line detection schemes initially developed for chromatography or capillary electrophoresis (spectroscopy, conductimetry…). The implementation of such an in-line (or even on-chip analysis) should pave the way to real time dosimetric measurements.

The system presents, however, an apparent limitation due to the bulging of the ultrathin Si_3_N_4_ membrane, which increases the thickness of the irradiated water film. Indeed, it is difficult to envision that all users could have access to an optical profilometer to characterize the membrane geometry under flow. This problem will probably have a technical solution in the near future, as membranes stabilized by a pillar array (Creemer *et al.*, 2011 ▸), which exhibit low deformation under flow, are now commercially available [NanoInsight, Delft, The Netherlands (https://www.nanoinsight.nl/)]. One should note, though, that deformation only impacts dosimetry measurements when the X-ray attenuation length in water is about the same or larger than the nominal thickness of the water film. Our dosimetry experiments clearly falls into this category (see Fig. S8) as it corresponds to an attenuation length at 1.28 keV of 4.7 µm, very close to the nominal film thickness of 5.3 µm, and to a transmission factor of 33% through the 5.3 µm water film.

By contrast, when the attenuation length is much smaller than the nominal thickness, the X-ray beam is almost completely absorbed by the water film in the absence of bulging. Therefore bulging does not affect dose measurement as the dose is mostly deposited close to the irradiation window. To be in this bulging-independent regime, one should make sure that the 5.3 µm-thick film absorbs more than 95% of the beam, which corresponds to an attenuation length smaller than 1.75 µm or, equivalently, to a mass absorption coefficient of water, 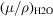, higher than 5 × 10^3^ cm^2^ g^−1^. Such values are achieved at photon energies below 270 eV, or in the 550–880 eV interval (Henke *et al.*, 1993 ▸). Increasing the nominal microchannel thickness is a way to reach this bulging-independent regime for other energy values, although at the cost of a dilution of the radiolytic products that may be problematic at very low dose.

For energies with large attenuation lengths in water (*i.e.* in the hard X-ray domain), one can use thicker membranes to limit the bulging and reach the electronic equilibrium in the medium which ensures a proper dosimetric measurement (Carlsson, 1985 ▸). For example, using 3 µm-thick Si_3_N_4_ windows will limit the deformation to 1/20th of the one observed here (Martins *et al.*, 2009 ▸), *i.e.* to less than 350 nm, while allowing to work with X-rays down to 10 keV [their secondary electrons have ranges of about 1.5 µm in Si_3_N_4_ (Berger *et al.*, 2005 ▸)]. Under these conditions, the dosimetric water film is almost transparent to X-rays (transmission > 0.997), while capturing enough dose (about 1 Gy at 10 keV and 1  ×  10^10^ photon s^−1^) at low flow rate (3 µL min^−1^) to allow measurements using a fluorescent dosimeter of the benzoate/coumarin family. The system presented here should therefore be applicable to intermediate and hard X-ray beamlines, provided the *G*-values in this energy range are known.

The cumulated errors (15%) during *G* calibration at 1.28 keV may appear substantial. However, many of them are related to the need to measure the transmissions (*T*_air_, *T*_Si3N4_, *T*_solution_) and to align the cell using a photodiode. A straightforward improvement would therefore be to transfer the cell under vacuum (no more *T*_air_), and to integrate a second Si_3_N_4_ membrane at the back of the microfluidic cell, so as to allow direct measurement of the transmission values (*T*_Si3N4_ and *T*_solution_) thanks to a photodiode located just behind the cell. This should reduce the total errors below 8% for a submillimetric irradiation field at 1.28 keV. This latter value compares well with the metrological errors determined in liquid Fricke dosimetry for centimetric irradiation field (3–4%), keeping in mind that these errors actually increase when the irradiation field size decreases (Austerlitz *et al.*, 2006 ▸). Owing to these errors, the use of liquid dosimeters to map irradiation fields have indeed been limited to macroscopic ones. This is the first time that a liquid dosimeter is used not only to measure locally but also to map the irradiation field in water on a submillimetre scale. Both the precision on dosimeter reading and the spatial resolution achieved (15 µm in Fig. 5 ▸) are significantly higher than those obtained with a hydrogel dosimeter using magnetic resonance imaging [300% errors, 200 µm resolution (Bräuer-Krisch *et al.*, 2015 ▸)]. Moreover, because the dosimetric medium is constantly renewed, the microfluidic dosimetry device presented here does not suffer the beam damage limitation inherent to solid state devices used for microbeam mapping (Archer *et al.*, 2019 ▸). Therefore, it could advantageously be adapted to allow full-field imaging of the photon field generated by the complex beam profiles used in synchrotron microbeam radiation therapy (MRT).

## 5. Conclusion   

With the dawn of fourth-generation synchrotrons, the need for characterization and control of the deposited dose will become essential in order to prevent radio-induced damage, especially to biological samples. We have demonstrated that the developed microfluidic dosimetry cell is perfectly adapted to synchrotron micro-beam dosimetry and that the dose delivered to the sample upon soft X-ray exposure can be measured with reasonable accuracy. In particular, the determined *G*(HO^**.**^) value of 0.025 ± 0.004 µmol J^−1^ found at 1.28 keV has shown great robustness and confirms the high linear energy transfer behavior of soft X-rays. In the near future, the implementation of the microfluidic cell directly under vacuum will maximize the dose deposited in the sample, by suppressing the air absorption contribution, a limiting factor at low photon energy. This will allow studying the effect of core-ionization, such as the carbon *K*-shell ionization (290 eV), on liquid biological samples.

## 6. Related literature   

The following references, not cited in the main body of the paper, have been cited in the supporting information: Fonin *et al.* (2014 ▸); Gu & Kenny (2009 ▸); Zhadin & Alfano (1998 ▸).

## Supplementary Material

Supporting information file. DOI: 10.1107/S1600577521002691/ve5141sup1.pdf

## Figures and Tables

**Table 1:** Non-exhaustive list of common chemical dosimeters

Dosimeter	Chemical change	Method of measurement	Dose range (Gy)	References
Solid dosimeter				
Radiochromic films	Polymerization	Densitometry	10–1000	(Crosbie *et al.*, 2008 ▸)
Plastic scintillators		Light emission	0.01–1000	(Beddar *et al.*, 1992 ▸)

Liquid dosimeter				
Aqueous ferrous sulfate	Fe^2+^ → Fe^3+^	Spectrophotometry	20–400	(Fricke & Hart, 1935 ▸)
Aqueous ceric sulfate	Ce^4+^ → Ce^3+^	Spectrophotometry	100–10^6^	(Hardwick, 1952 ▸)
Aqueous polyacrylamide	Polymer degradation	Viscosity	0.5–75	(Boni, 1961 ▸)
Benzoate	Hydroxylation	Fluorimetry	0.05–200	(Armstrong *et al.*, 1960 ▸)
Coumarin	Hydroxylation	Fluorimetry	0.01–60	(Ashawa *et al.*, 1979 ▸)
Rhodamin B	Photo-inactivation	Spectrophotometry	100–1900	(Beshir *et al.*, 2014 ▸)

**Table 2:** Uncertainties of the production yield of both 2HB and HO^**.**^ Values given for a 0.01 *M* benzoate solution irradiated with a 1.28 keV energy beam but can vary with photon energy or benzoate concentration.

Source	Uncertainty (%)
Photon flux measurement (φ_photodiode_)	3.3
Transmission of photodiode-to-membrane air gap (*T*_air_)	2.2
Centering of the beam on the photodiode and the membrane	5.6
Transmission of the silicone nitride membrane (*T*_Si3N4_)	1.4
Flow rate measurement	5
Transmission of the solution irradiated at 8 µL min^−1^ (*T*_solution_)	10†
Chemical dosimeter calibration	6.25†
**Total error**	**≤15**

† Increases with the concentration.

**Table 3:** *G*(HO^**.**^) values in water determined upon exposure to different radiations (time scale ≥ 100 ns, typical value for chemical scavenging processes)

Irradiation source	LET (eV nm^−1^)	*G*(HO^**.**^) (µmol J^−1^)	Dosimeter	References
^60^Co Gamma	0.2†	0.29	Benzoate	(Mozumder, 1999 ▸)
		0.27	Coumarin	(Ashawa *et al.*, 1979 ▸)
20.9 MeV Deuteron	4.5	0.196	Formic acid	(Anderson & Hart, 1961 ▸)
20 GeV Ar^18+^ ions	92	0.022	Luminol	(Baldacchino, 2015 ▸)
Al *K*-shell X-rays 1.49 keV		0.072	DNA plasmids	(Fulford *et al.*, 1999 ▸)
Synchrotron X-rays 1.28 keV		0.038 ± 0.003	Benzoate	(Huart *et al.*, 2020 ▸)
Synchrotron X-rays 0.4 keV		0.092 ± 0.018	Benzoate	(Huart *et al.*, 2020 ▸)
Synchrotron X-rays 0.3-0.6 keV	520†	0.023 ± 0.003	Therephthalic acid	(Vyšín *et al.*, 2020 ▸)
5 MeV He^2+^ ion	200	0.049	Formic acid	(Anderson & Hart, 1961 ▸)
18.3 MeV/nucleon carbon	230	0.035	Phenol	(LaVerne, 1989 ▸)

† LET of secondary electrons.
