# Supplementary material for: A microfluidic dosimetry cell to irradiate solutions with poorly penetrating radiations: a step towards online dosimetry for synchrotron beamlines
Source: J Synchrotron Radiat. 2021 Apr 19;28(Pt 3):778–89. doi: 10.1107/S1600577521002691 (PMC8127378; doi:10.1107/S1600577521002691)
Supplement: Supplementary file 1 [file s-28-00778-sup1.pdf]

JOURNAL OF SYNCHROTRON RADIATION

Volume 28 (2021)

Supporting information for article:

A microfluidic dosimetry cell to irradiate solutions with poorly penetrating radiations: a step towards online dosimetry for synchrotron beamlines

Lucie Huart, Christophe Nicolas, Marie-Anne Hervé du Penhoat, Jean-Michel Guigner, Charlie Gosse, Jérôme Palaudoux, Stephane Lefrançois, Pascal Mercere, Paulo Dasilva, Jean-Philippe Renault and Corinne Chevallard

Fig. S1: Calibration curves for the fluorescent signal emitted by sodium 2-hydroxybenzoate (Na_2HB) diluted in sodium benzoate solutions (NaBz) at different concentrations: light to dark blue correspond to 0.01 M, 0.1 M and 1 M. Experimental data were fitted to a straight line of equation $F = F_1 * [Na2HB] + F_0$. Excitation at 300 nm and emission is accorded at 420 nm. Only statistical uncertainties are shown. The lower slope observed on the highly concentrated solution (1 M) is attributed to an inner-filter effect (Fonin 2014, Gu 2009, Zhadin 1998).

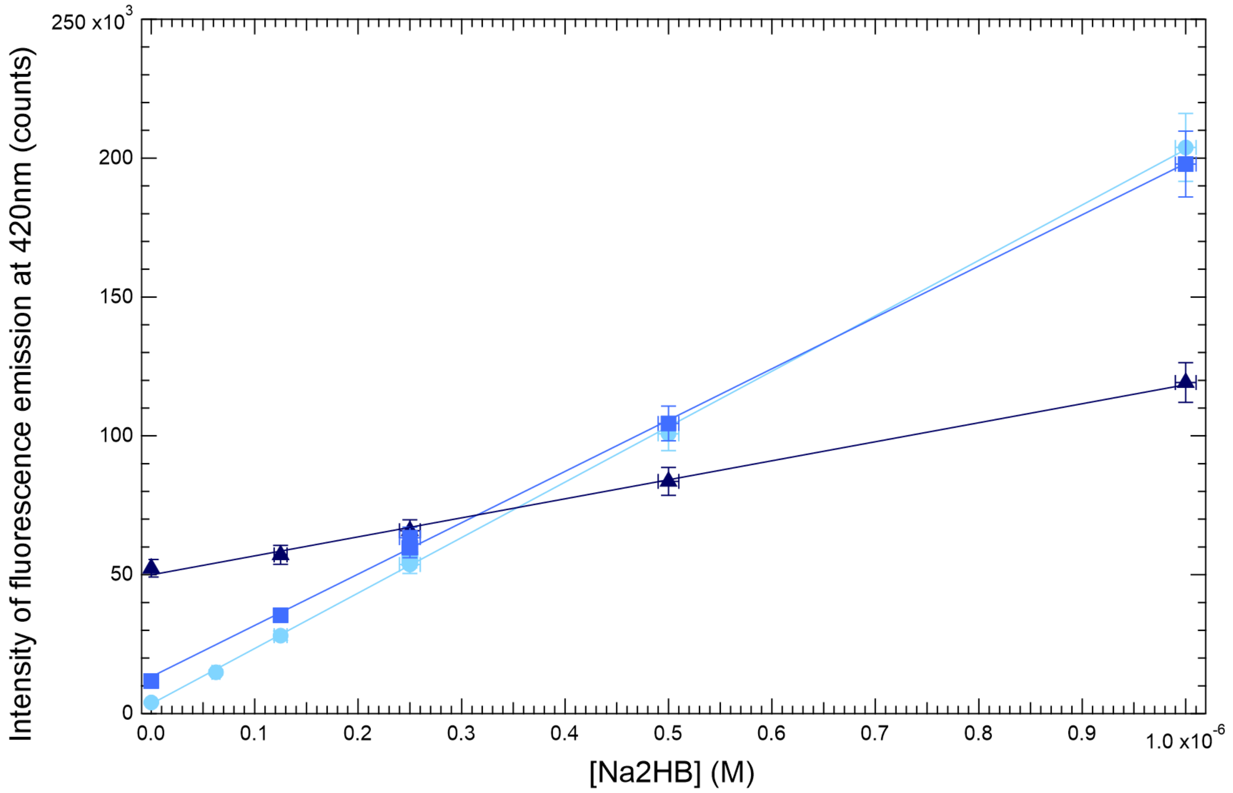

[NaBz] (M)	F_1 (counts / M)	F_0 (counts)	R^2
0.01 M	*2.05E+11 ± 1.28E+10*	*2.24E+03 ± 1.66E+03*	0.999495
0.1 M	*1.90E+11 ± 1.60E+10*	*1.22E+04 ± 3.55E+03*	0.999099
1 M	*7.06E+10 ± 6.07E+09*	*4.83E+04 ± 1.32E+03*	0.996583

Fig.S2:Diagram of the chips assembled to form the microfluidic cell (not to scale). A- Front chip including the window for irradiation, B-Back, including the fluidic inlet and outlet. Left, top view; right cross-section views

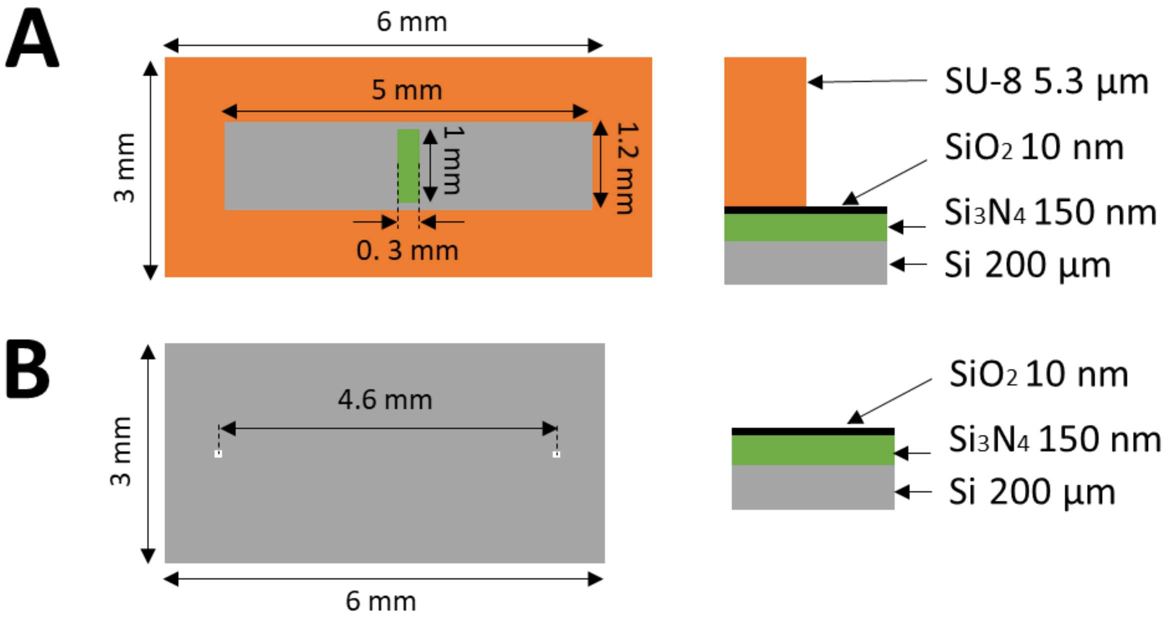

Fig. S3: Measurement of the height of the SU-8 spacer (H_{Spacer}) by interferometry. Average profile was plotted in the x-z plane and led to a value for the microfluidic channel height of H_{spacer}= 5.3 µm.

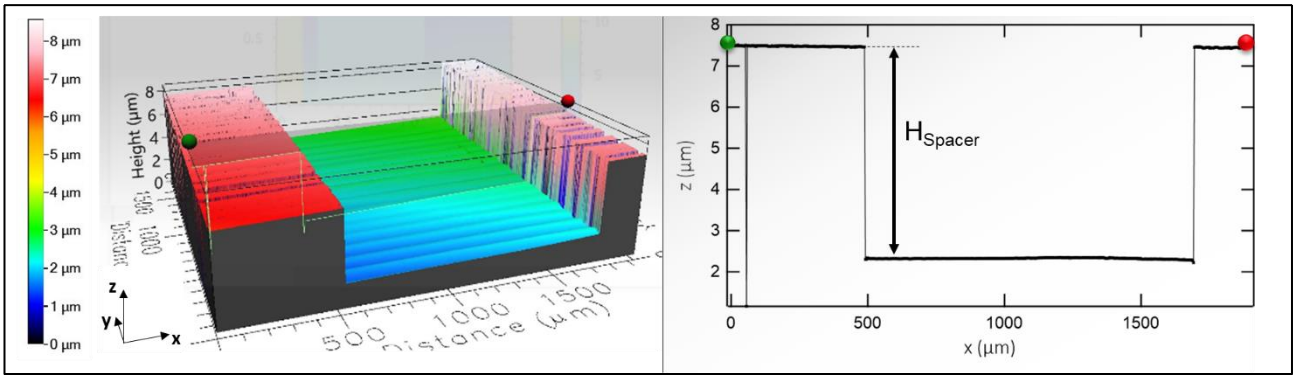

Fig. S4: Vertical scan of the beam on the masked photodiode (black symbols) with an error of 2% on the intensity measurement. A fit of the experimental data (red line) was performed by convolving a rectangular function (blue curve), 1.07 mm wide (so as to mimic the z-dimension of the mask) and a Gaussian function (grey dotted line) corresponding to the vertical beam profile. The vertical width of the beam, determined this way, is

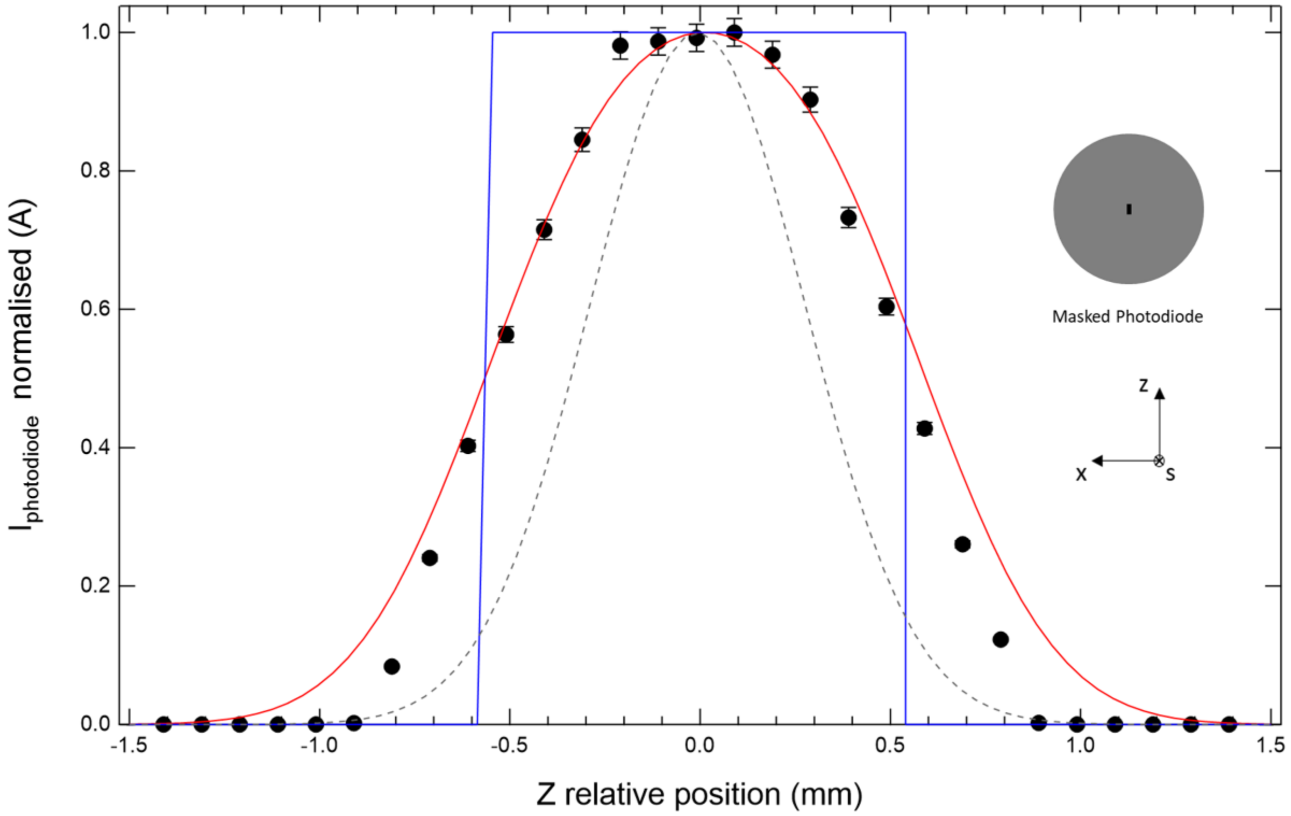

Fig. S5- Current measured at the masked photodiode ($I_{photodiode}$) as a function of the dimension of the beamline monochromator exit slit.

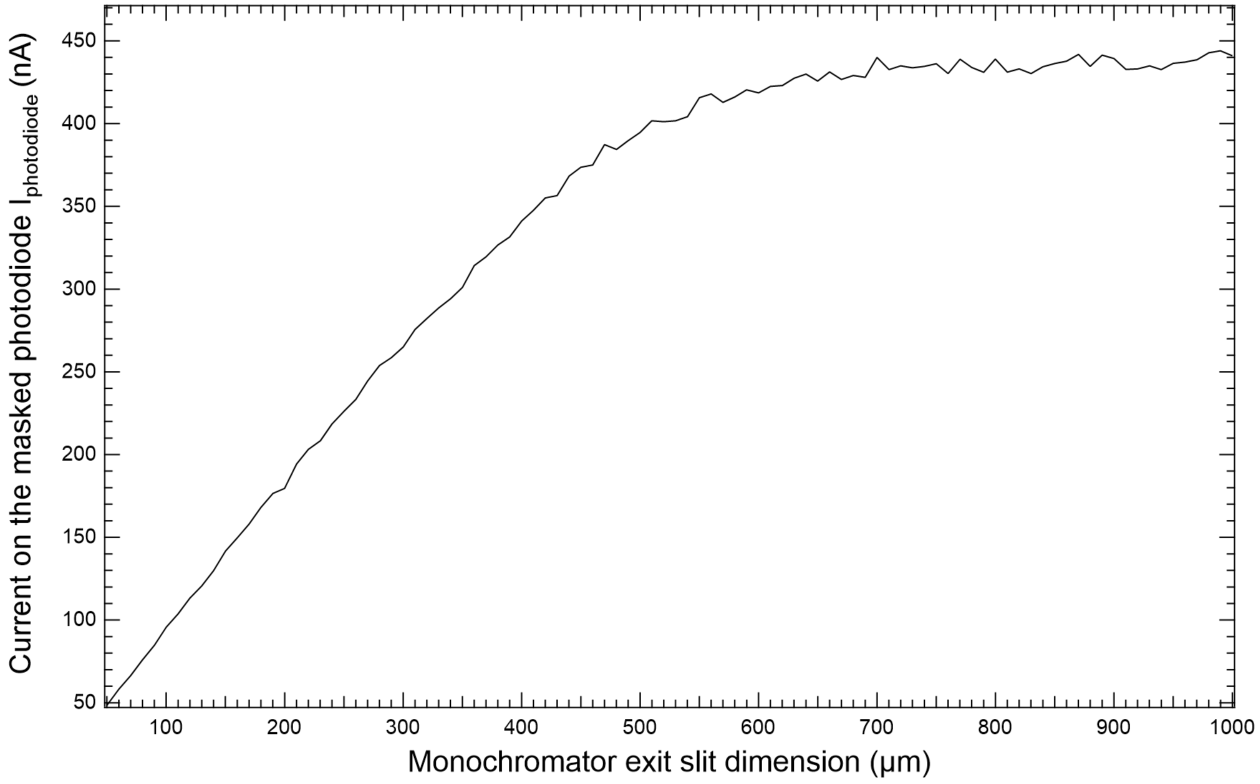

Fig. S6: (A) Flowrate (Q) reported by upstream/downstream flowmeters as a function of the actuation pressure difference (ΔP). Results are presented for different sodium benzoate concentrations: light to dark blue correspond to 0.01 M, 0.1 M and 1 M. Only statistical errors are shown. Experimental data are fitted to straight lines of equation:

$\Delta P = R_{tot} * Q$, where R_{tot} is the total hydrodynamic resistance of the fluidic system.

(B) Experimental viscosity measured by an Oswald viscosimeter (error of 0.006 mPa.s) plotted as function of the deduced hydrodynamic resistivity R[illegible] for different concentrations of benzoate. A linear fit conducted to the

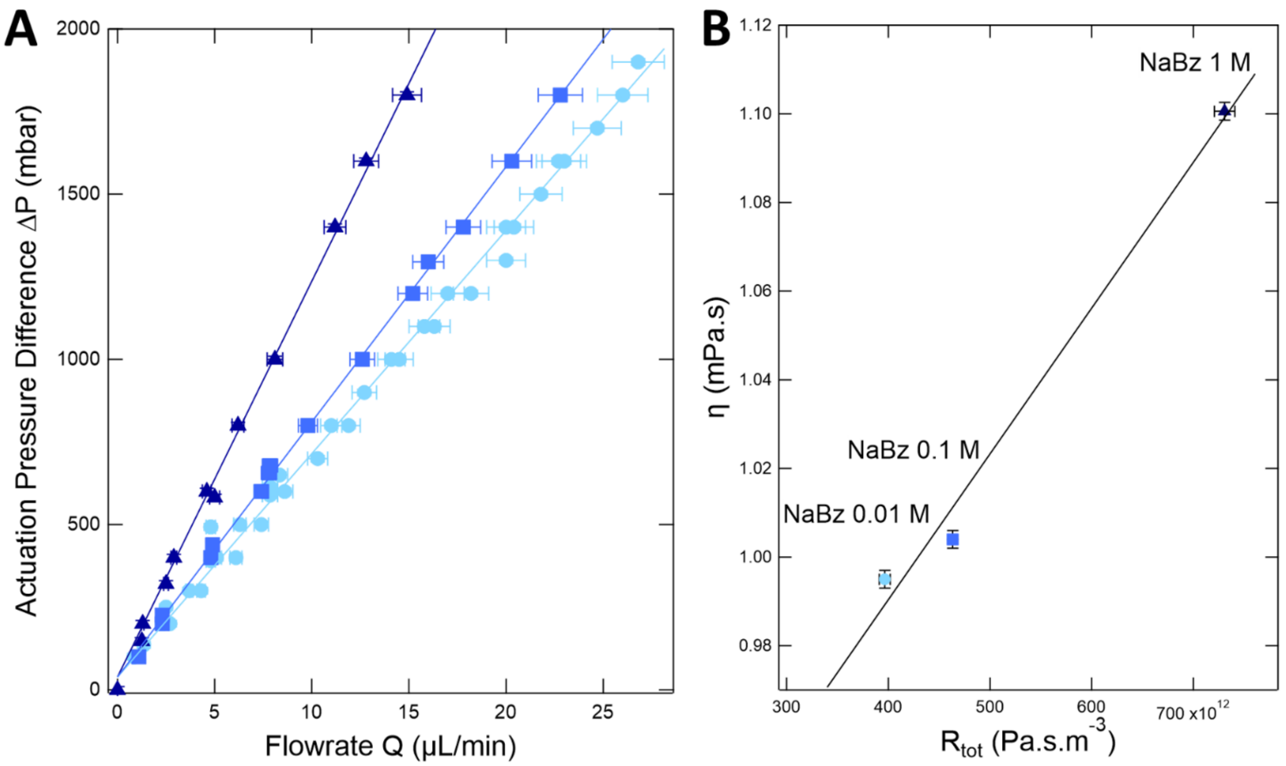

Fig. S7: Deformation of the Si_3N_4 membrane measured along its width by interferometry. Results are presented for different values of the actuation pressure difference ΔP. Experimental data (black dots) are fitted to parabolas (red lines).

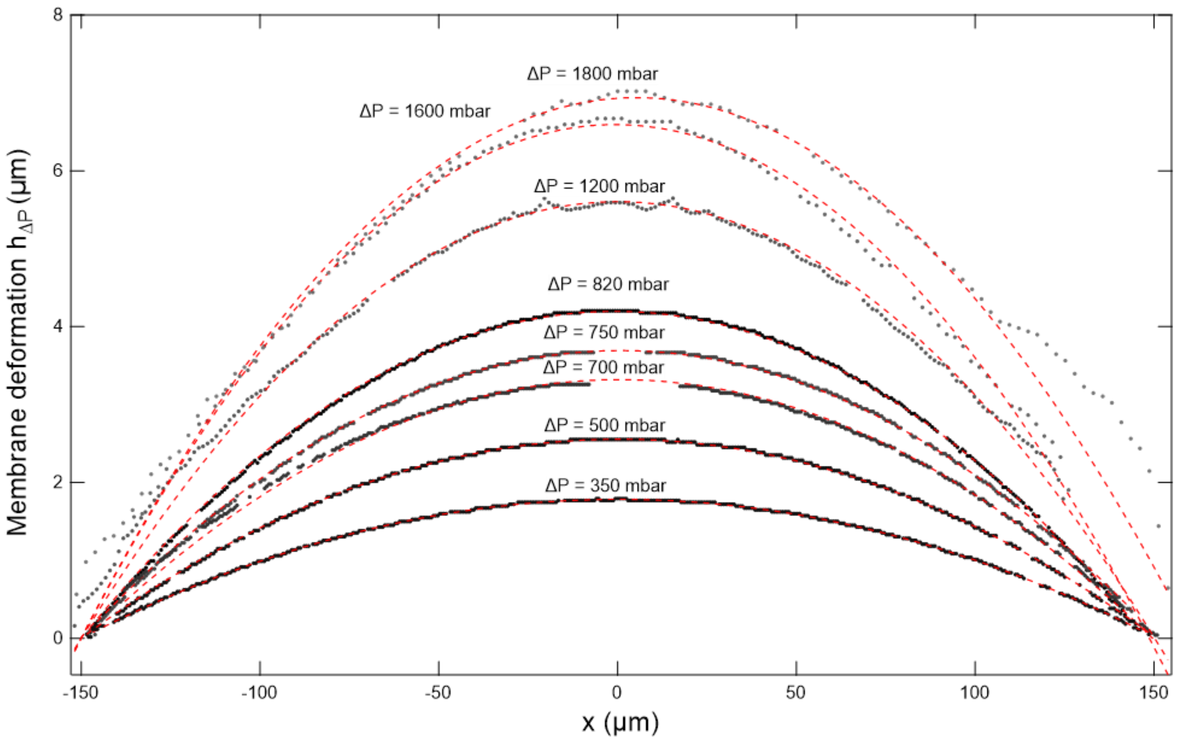

Fig. S8: Computed evolution of the absorption coefficient of the dosimeter solution at 1.28 keV, as function of the sample thickness in the 5.3 to 12.5 µm range. Calculations have been performed based on the elemental linear absorption coefficients provided by the National Institute of Standards and Technology (NIST) (Henke 1993).

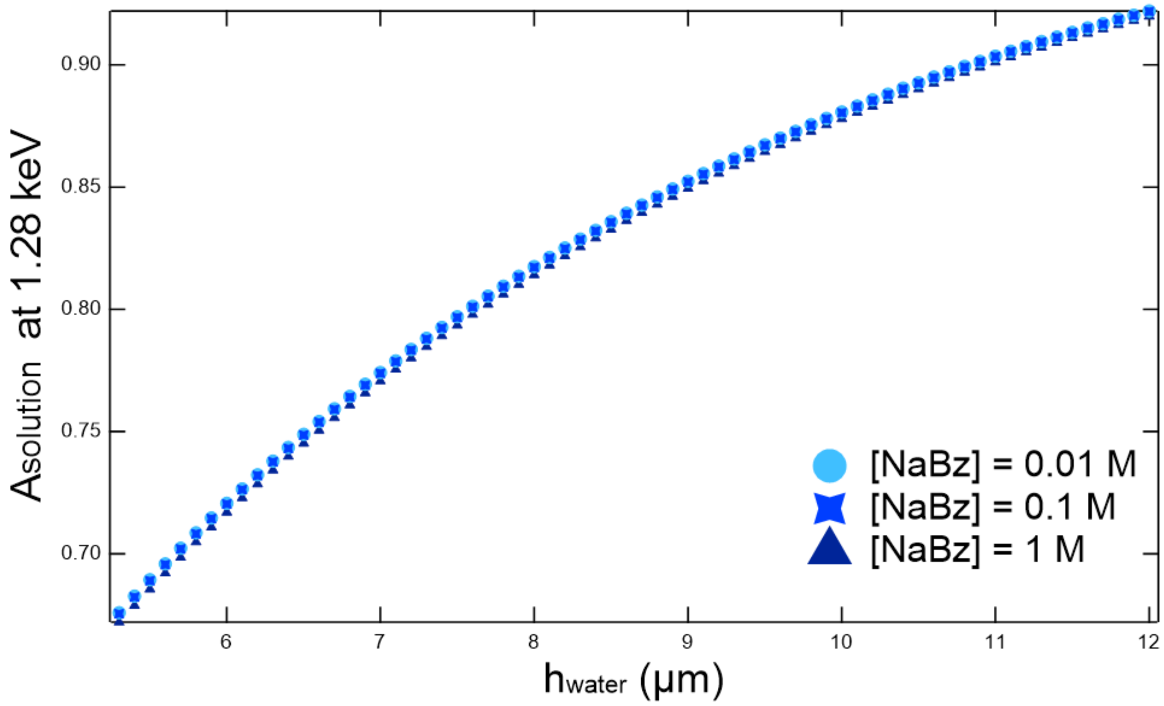

Fig. S9: Relationship between the absorbed dose and 2-hydroxybenzoate (2HB) concentration in 0,01M NaBz solutions irradiated at 1.28 keV X-rays. Measurements carried out in 2019 with a different batch of benzoate. Concentration of 2HB was determined with fluorescence measurements as described in the Methods section of the article. Resulted G(OH°) is

0.025 ± 0.004 µmol/J.

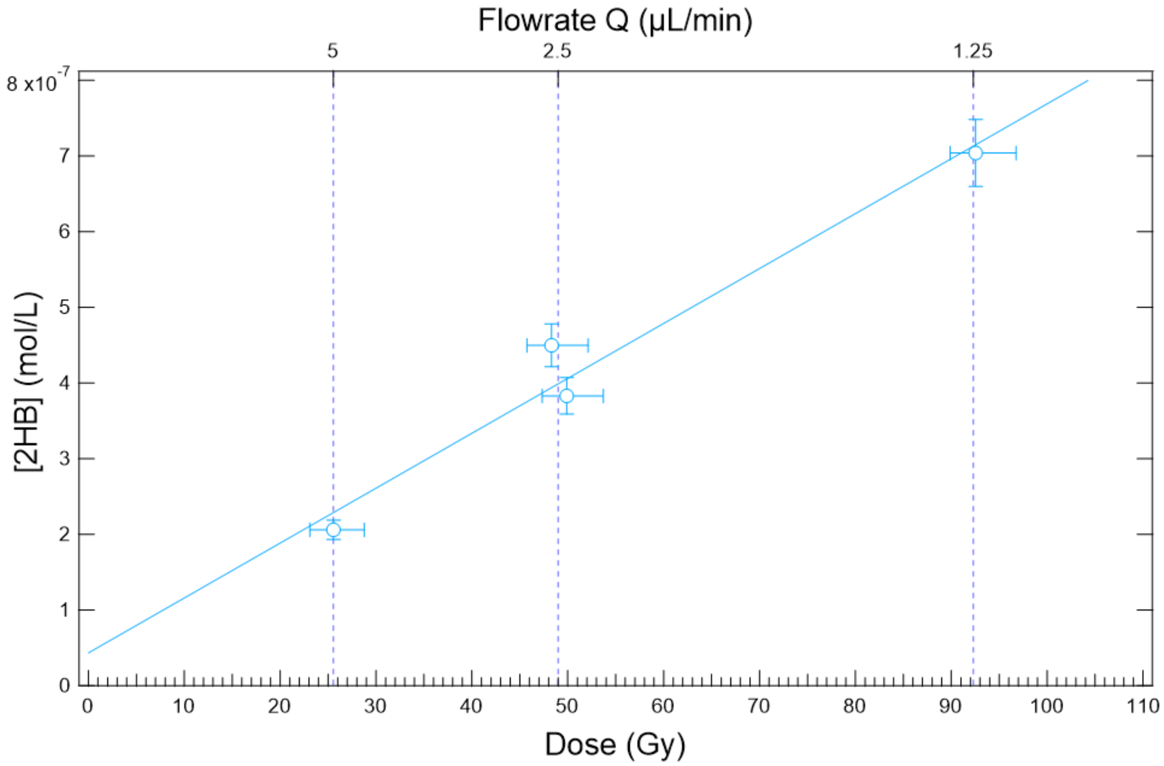

References :

Fonin, A. V., Sulatskaya, A. I., Kuznetsova, I. M., & Turoverov, K. K. (2014). Fluorescence of dyes in solutions with high absorbance. Inner filter effect correction. *PLoS ONE*, *9*(7). https://doi.org/10.1371/journal.pone.0103878

Gu, Q., & Kenny, J. E. (2009). Improvement of inner filter effect correction based on determination of effective geometric parameters using a conventional fluorimeter. *Analytical Chemistry*, *81*(1), 420–426. https://doi.org/10.1021/ac801676j

Zhadin NN, Alfano RR. Correction of the internal absorption effect in fluorescence emission and excitation spectra from absorbing and highly scattering media: theory and experiment. J Biomed Opt. 1998 Apr;3(2):171-86. doi: 10.1117/1.429874. PMID: 23015054.
